# Comprehensive insights of pretreatment strategies on the structures and bioactivities variation of lignin-carbohydrate complexes

**DOI:** 10.3389/fbioe.2024.1465328

**Published:** 2024-08-20

**Authors:** Chen Su, Xiu Wang, Yongjun Deng, Zhongjian Tian, Chen Huang, Guigan Fang

**Affiliations:** ^1^ State Key Laboratory of Biobased Material and Green Papermaking, Qilu University of Technology, Shandong Academy of Sciences, Jinan, China; ^2^ Institute of Chemical Industry of Forest Products, Chinese Academy of Forestry, Nanjing, China; ^3^ Co-Innovation Center for Efficient Processing and Utilization of Forest Resources, Nanjing Forestry University, Nanjing, China; ^4^ Key Laboratory of Polymer Chemistry and Physics of Ministry of Education, School of Materials Science and Engineering, Peking University, Beijing, China

**Keywords:** lignin-carbohydrate complex, pretreatment methods, structure variation, antioxidant, anti-ultraviolet

## Abstract

**Introduction:** Due to its unique structural features and bioactivities, the lignin-carbohydrate complex (LCC) displays great potential in vast industrial applications. However, the elucidation of how various pretreatment methods affect the structure and bioactivities remains unaddressed.

**Method:** The three pretreatment methods were systematically studied on the variations of structures and bioactivities, and the Gramineae plant, i.e., wheat straw, was adopted in this study. The structures and bioactivities variation caused by different pretreatments were studied in detail.

**Result and Discussion:** The results showed that compared to physical or chemical pretreatments, biological pretreatment was the most effective approach in improving the bioactivities of LCC. The LCC from biological pretreatment (enzymatic hydrolysis, ELCC4) had more functional groups while the lower weight-average molecular weight (*Mw*) and polydispersity index (PDI) were well-endowed. The highest antioxidant abilities against ABTS and DPPH of ELCC4 were high up to 95% and 84%, respectively. Furthermore, ELCC4 also showed the best ultraviolet (UV)-blocking rate of 96%, which was increased by 6% and 2% compared to LCC8 (physical pretreatment) and LLCC4 (chemical pretreatment). This work prospectively boosts the understanding of pretreatment strategies on the structures and bioactivities variation of LCC and facilitates its utilization as sustainable and biologically active materials in various fields.

## 1 Introduction

Lignin-carbohydrate complex (LCC), an integral component of plant cell walls, is renowned for its complex structures and multifaceted bioactivities (Zhang and Naebe, 2021; Ullah et al., 2022; Wang R. et al., 2024). LCC possesses a unique blend of structural features that endow them with antioxidant, antimicrobial, and antiultraviolet properties (Sakagami et al., 2010; Tarasov et al., 2018; Wang X. et al., 2024). Such attributes render LCC highly promising for a broad application spectrum, ranging from pharmaceuticals to cosmetics (Giummarella et al., 2019). Despite their vast potential, the industrial exploitation of LCC has been hindered due to the lack of clarity regarding the impact of current isolation procedures on their structures and bioactivities. It is important to understand the effects of various pretreatments on the structures and bioactivities transformation for accelerating the utilization of LCC in various fields.

Currently, three pretreatment methods, including physical (Du et al., 2013a), chemical (Singh et al., 2005; Bao et al., 2024), and biological strategies (Huang et al., 2019), are the principal approaches to manipulating the structure of LCC. The chemical bonds and molecular interactions within LCC could be regulated through different pretreatments thereby influencing their biological responses. For instance, physical pretreatment, such as high-energy milling, can disrupt intermolecular forces, potentially enhancing solubility and reactivity (Gu et al., 2015). Chemical pretreatment, involving acids or bases, can cleave ester or ether linkages, altering the distribution of functional groups and possibly boosting antioxidant capacity (Su et al., 2021a; Wang et al., 2023). Biological pretreatment, mediated by enzymes or microbes, can selectively deconstruct lignin or polysaccharides, leading to the aromaticity and hydrophilicity changes of LCC, which may affect their antimicrobial properties (Narron et al., 2017).

Despite the individual insights gained from studying the effects of these pretreatment methods on LCC structure and bioactivity, a comprehensive evaluation that encompasses all three approaches is still lacking. Current research predominantly focuses on the isolated impacts of one or two pretreatment methods (Du et al., 2013b; Niu et al., 2016), neglecting the synergistic or antagonistic effects that might arise from a combinatorial approach. Furthermore, studies often draw conclusions based on LCC derived from disparate sources, introducing variability due to species-specific differences in LCC compositions and structures (Jiang et al., 2018; Huang et al., 2019). This heterogeneity complicates the establishment of generalizable principles governing the relationships between LCC pretreatments and bioactivities enhancement. A systematic and integrated assessment of the effects of physical, chemical, and biological pretreatments on LCC structure and bioactivity within a single species is paramount to bridge this knowledge gap and propel LCCs towards large-scale industrial implementation.

**FIGURE 1:**
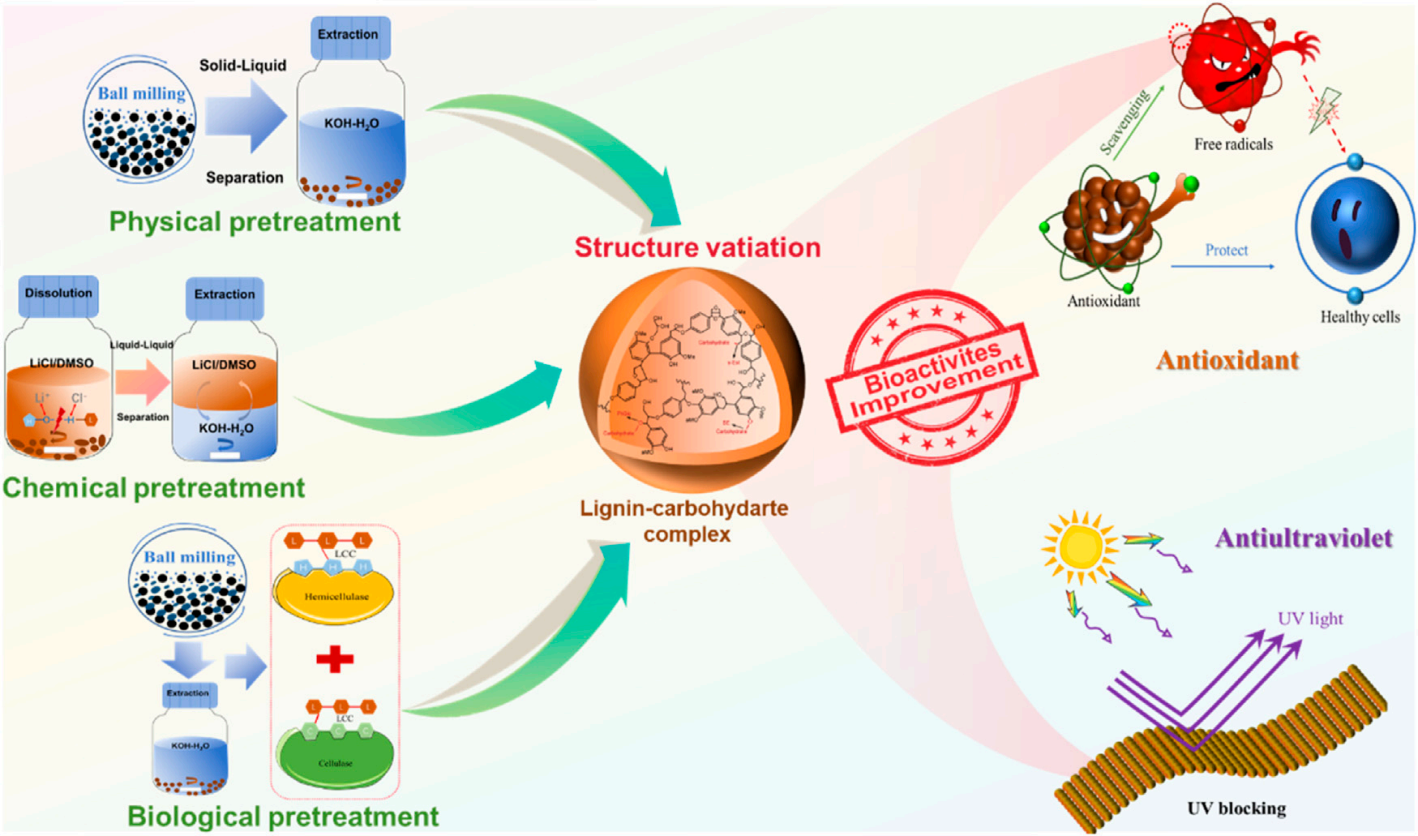
Schematic illustration of different pretreatment methods to fabricate lignin-carbohydrate complex (LCC) with antioxidant and ultraviolet (UV)--blocking properties.

Herein, three pretreatment methods were systematically studied on the variations of structures and bioactivities, and the Gramineae plant, i.e., wheat straw, was adopted in this study (Figure 1). The structures and bioactivities variation caused by different pretreatments were studied in detail. The results showed that the highest antioxidant abilities against ABTS and DPPH of ELCC4 (biological pretreatment) were high up to 95% and 84%, respectively. Furthermore, ELCC4 also showed the best ultraviolet (UV)-blocking rate of 96%, which was increased by 6% and 2% compared to LCC8 (physical pretreatment) and LLCC4 (chemical pretreatment). The goal of this research is to establish a robust foundation for the understanding of structure variation and bioactivities improvement of LCC using different pretreatment processes and expect to provide a feasible scheme for producing high bioactive LCC.

## 2 Materials and methods

### 2.1 Materials and chemicals

Wheat straw was collected from a local farm in Jurong (Jiangsu, China), and fractioned into stalks without internode. The air-dried wheat straw was milled to pass 30 mesh in a Wiley mill, and the dried powder was milled in a vibratory ball mill (QMQX, Nanjing Nanda Instrument, China) for 4 or 8 h. The resultant ball-milled wheat straw powder was chemically composed of 21.5% of Klason lignin, 1.7% of acid-soluble lignin, 37.2% of glucan, 19.1% of xylan, and 2.4% of arabinan. All chemicals, i.e., 2,2′-azino-bis (3-ethylbenzothiazoline-6-sulfonic acid) diammonium salt (ABTS), 2,2-diphenyl-1-picrylhydrazy (DPPH), cellulase (No. C2730), xylanase (No. X2753), DMSO, LiCl, KOH, etc., were purchased from Sigma-Aldrich.

### 2.2 Preparation of LCCs

20 g of ball-milled (4 h ball milling time) wheat straw powder was mixed with 10 wt% KOH solution (bio-mass to solution ratio was 1/4, w/w) and maintained for 24 h. Then, the resultant was centrifuged at 8,000 rpm, and the supernatant was dialyzed with de-ionized (DI) water for 72 h using a dialysis bag (T34-35-001, 3500 Da). Before rotary-evaporating the supernatant volume to 20 mL, 0.1 mol/L of HCl was utilized for precipitation and pH adjustment to 2.0. The precipitation was extracted with DMSO for 72 h to obtain the basic sample and named LCC4.

The physical pretreatment LCC (LCC8) was fabricated by prolonging the ball milling time to 8 h, and the other procedures were the same as the LCC4.

For the LCC prepared from chemical pretreatment (LLCC4), 20 g of ball-milled (4 h ball milling time) wheat straw powder was mixed with 250 g of LiCl/DMSO (8/92, w/w) solution and stirred at room temperature for 24 h, which was based on previous works (Liu et al., 2016; Gan et al., 2021). The mixture was subsequently dropwise added into a 10 wt% KOH solution (bio-mass to solution ratio was 1/4, w/w) and maintained for 24 h. Then, the resultant mixture was centrifuged at 8,000 rpm, and the supernatant was dialyzed with DI water for 72 h using a dialysis bag (T34-35-001, 3500 Da). Before rotary-evaporating the supernatant volume to 20 mL, 0.1 mol/L of HCl was utilized for precipitation and pH adjustment to 2.0. The precipitation was extracted with DMSO for 72 h to obtain LLCC4.

The biological pretreatment LCC (ELCC4) was fabricated after enzymatic hydrolysis of LCC4. Briefly, LCC4 was transformed in a 125 mL Erlenmeyer flask containing enzymatic solution (LCC4 to solution ratio was 5/95, w/v), and placed in a shaking bed incubator (Model THZ-98C, Yi Heng, Shanghai) at 200 rpm and 50°C for 12 h. The enzymatic solution was composed of cellulase (20 FPU/g glucan), xylanase (140 IU/g glucan), and 50 mM citrate buffer (pH 4.8). Subsequently, the reactant was centrifuged at 5,000 rpm for 20 min, and the precipitate was collected and washed with DI water three times. After freeze-drying, the ELCC4 was obtained. For convenience, LCCs obtained from different pretreatments were labeled with abbreviations, as shown in Table 1.

**TABLE 1:** Abbreviations of LCCs obtained from different pretreatments.

Treatment type	Approach	Abbreviation
—	Separate 4 h ball-milled wheat straw by solid-liquid (KOH-H_2_O) separation	LCC4
Physical	Separate 8 h ball-milled wheat straw by solid-liquid (KOH-H_2_O) separation	LCC8
Chemical	Separate 4 h balled-milled wheat straw by liquid-liquid (LiCl-DMSO/KOH-H_2_O) separation	LLCC4
Biological	Enzymatic hydrolysis LCC4 for 12 h	ELCC4

### 2.3 Characterizations

The chemical compositions of preparations were determined using a conventional two-step acidolysis (Su et al., 2021a; Su et al., 2024). Briefly, the samples were first hydrolyzed using sulfuric acid in two stages. The hydrolysis conditions were an acid concentration of 72% (v/v) at 30°C and 3.6% (v/v) at 120°C for the first and second stages, respectively. The hydrolysis duration time was 1 h for both stages. The hydrolysate was then analyzed for carbohydrates using an improved high-performance anion-exchange chromatographic method using pulsed amperometric detection (HPAEC-PAD). The Klason lignin content was measured gravimetrically after washing and drying the solid residue from the acid hydrolysis. The acid-soluble lignin content was measured by UV-vis spectra.

The *Mw*, number-average molecular weight (*Mn*), and PDI (*Mw*/*Mn*) of samples were analyzed using gel permeation chromatography (GPC, LC-20A, Shimadzu Co., Japan) with an RID. The thermogravimetric (TG) and differential thermal (DTG) measurements of LCCs were conducted on a thermal analyzer (TG 209 F1 libra, Netzsch, Germany). The heating rate was fixed at 10 K/min, and the testing temperature was performed from 30°C to 800°C under a dry nitrogen atmosphere (Wang X. et al., 2021; Wang et al., 2022; Su et al., 2024). ^13^C NMR, ^31^P NMR, and 2D ^1^H-^13^C heterogeneous single quantum correlation (HSQC) NMR of different specimens were analyzed using a Bruker AVANCE 600 MHz spectrometer. For ^13^C NMR, 100 mg of preparation was dissolved in 0.5 mL DMSO-*d*_*6*_ solution, then added into 40 μL 0.01 M of chromium (Ⅲ) acetylacetonate for testing. For ^31^P NMR analysis, 20 mg of the sample was dissolved in 0.5 mL anhydrous pyridine-*d*_*5*_/CDCl_3_ (1.6/1, v/v). 100 μL of cyclohexanol (11.02 mg/mL, internal standard) and 100 μL chromium (Ⅲ) acetylacetonate (5 mg/mL, relaxation regent) prepared using anhydrous pyridine-d_5_/CDCl_3_ solution were mixed with the sample solution and added to 60 μL phosphitylating regent (2-chloro-4,4,5,5-tetramethyl-1,2,3-dioxaphospholane), with constantly stirring at room temperature for 30 min, and then tested all sample immediately in 30 min. For HSQC NMR measurement, 70 mg of preparation was dissolved in 0.5 mL of deuterated dimethyl sulfoxide (DMSO-*d*_*6*_), as described previously (Chen et al., 2017; Su et al., 2021b; Cai et al., 2023).

### 2.4 Bioactivities determination

The antioxidant activities were evaluated with the radical scavenging capacity of DPPH and ABTS using a spectrophotometric method (Xie et al., 2020; Su et al., 2021c; Liang et al., 2023). The concentrations of samples were varied from 0.05 to 1.5 mg/mL. Ferric reducing antioxidant power (FRAP) assay was described in previous work (Gan et al., 2021). Briefly, LCC solutions (dissolved in DMSO) with different concentrations (0.05–1.5 mg/mL) were mixed with 2.5 mL of phosphate buffer (0.2 M, pH = 6.6) and 2.5 mL of potassium ferricyanide [K_3_Fe(CN)_6_] (1.0%, w/v) solution respectively. After the reaction at 50°C for 20 min, 2.5 mL of trichloroacetic acid (TCA) (10%, w/v) was added and the mixture was centrifuged (3,000 rpm, 10 min). Later, 2.5 mL of supernatant was mixed with 0.5 mL of ferric trichloride (FeCl_3_) (0.1%, w/v) and 1 mL of distilled water to terminate the reaction. Finally, its UV absorbance was measured at a wavelength of 700 nm after 10 min.

For UV-blocking measurements, the preparations were added to the DMSO solution and kept stirring till the sample dissolved completely. The mixture was then diluted to 0.5 mg/mL before testing. The UV absorbance in each sample was measured by Metash UV-8000 (Metash Instrument, Shanghai). The transmittance (T) of UVA, UVB, and UV (T_UVA_ (320–400 nm), T_UVB_ (280–320 nm), and T_UV_ (200–400 nm) of the samples were calculated according to Equations 1–3 (Bian et al., 2021; Su et al., 2024):

$$T_{\text{UVa}}=\frac{\int_{320}^{400}T_{\lambda }{\times d}_{\lambda }}{\int_{320}^{320}d_{\lambda }}$$ (1)

$$T_{\text{UVB}}=\frac{\int_{280}^{320}T_{\lambda }{\times d}_{\lambda }}{\int_{280}^{320}d_{\lambda }}$$ (2)

$$T_{\text{UV}}=\frac{\int_{200}^{400}T_{\lambda }{\times d}_{\lambda }}{\int_{200}^{400}d_{\lambda }}$$ (3)

where T_λ_ is the average spectral transmittance, d_λ_ is the bandwidth, and λ is the wavelength. The blocking rates for UVA, UVB, and UV were calculated according to Equations 4–6 (Zhang and Naebe, 2021; Su et al., 2024):

$$\text{UVA blocking rate}=1-T_{\text{UVA}}$$ (4)

$$\text{UVB blocking rate}=1-T_{\text{UVB}}$$ (5)

$$\text{UV blocking rate}=1-T_{\text{UV}}$$ (6)

## 3 Results and discussion

### 3.1 Physicochemical properties

**FIGURE 2:**
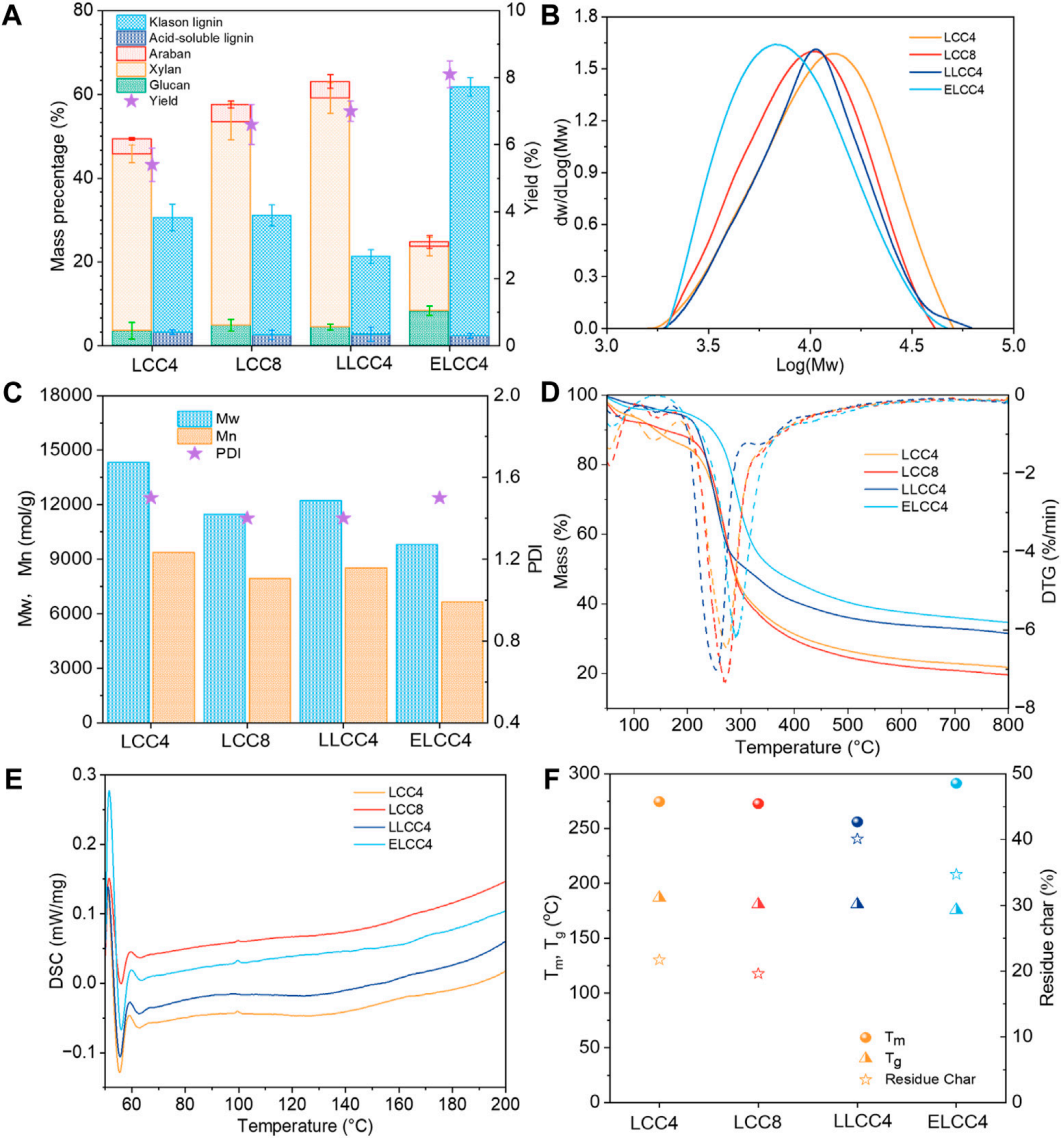
**(A)** Chemical composition, **(B)** weight-average molecular weight (*Mw*) distribution, **(C)** polydispersity index (PDI), **(D)** TG and DTG curves, **(E)** DSC curves, and **(F)** the maximum decomposition temperature (*T*_*m*_) and residue char (RC) of various LCCs.

The chemical composition plays a critical role in affecting the bioactivities. As shown in Figure 2A, the xylan with a proportion over 50% was the primary polysaccharide in LCCs. However, the lignin content of ELCC4 significantly increased to 60% while the carbohydrates decreased after enzymatic hydrolysis, indicating the effective hydrolysis of carbohydrates. This result was beneficial to exposing more bioactive groups in ELCC4. Noticeably, prolonging the balling time could barely change the composition compared to LCC4 and LCC8, which was also closely related to the bioactivities (Figure 2A), just affected yield. Contrarily, different separations have significantly impacted the composition of LCC even under the same ball milling time. The mass balance of LCC was not achieved 100%, probably due to the linkages between lignin and carbohydrates within the LCC preventing the hydrolysis of those carbohydrates linked with lignin, leading to discrepancies in the overall chemical composition. Overall, biological or chemical pretreatment can cleavage of chemical bonds between lignin and carbohydrates or within the lignin, leading to a relative variation of lignin and carbohydrate content. Physical pretreatment primarily disrupts the cellular structure of biomass materials through mechanical forces but usually does not significantly change the ratio of the lignin and carbohydrate.

All LCCs showed uniform *Mw* distribution with narrow PDI (<1.6, Figures 2B, C). The *Mw* of nonenzymatic LCCs were over 11,000 mol/g (Figure 2C), while this feature of ELCC4 greatly decreased to 9,800 mol/g (Figure 2C), signifying the successful hydrolysis of the polysaccharides. Compared with nonenzymatic LCCs, the slightly higher PDI of ELCC4 was attributed to the cutting off of the carbohydrate molecular chains during the enzymatic hydrolysis resulting in a wider *Mw* distribution (You et al., 2015). Due to the enzymatic hydrolysis, partial LC bonds within the ELCC4 were cleaved and their carbohydrate part would dissociate from the LCC, resulting in a varying in PDI. Moreover, the enzymatic time of ELCC4 was just 12 h, which was not enough to completely hydrolyze all carbohydrates of ELCC4, resulting in increasing the PDI value.

Biomaterials with good thermal stability possess a larger temperature range to release their bio-activities (Mohamad Ibrahim et al., 2011). In comparison to nonenzymatic LCCs (LCC4, LCC8, and LLCC4), ELCC4 exhibited excellent thermal stability as shown in Figures 2D, F, and the maximum decomposition temperature (*T*_*m*_) was high up to 296°C. The lignin fraction in ELCC4 was higher than that of nonenzymatic LCCs. However, the glass-transition temperature of ELCC4 was lower than that of nonenzymatic LCCs due to the lower *Mw* (Su et al., 2021b). The residue char (RC) of LLCC4 exceeded the other preparations (Figure 2F), because the S/G ratio of ELCC4 was lower than that of other LCC samples (Table 2), and S unit lignin had poorer thermal stability than G unit lignin (Zhou et al., 2021). Moreover, after LiCl-DMSO/KOH-H_2_O separation, the LLCC4 underwent the acid depolymerization reaction, forming more thermally stable carbon-carbon (C-C) bonds which increased the RC (Su et al., 2021a).

**TABLE 2:** Amount of lignin substructure of LCC preparations along with *Mw* and PDI.

Samples	LCC4	LCC8	LLCC4	ELCC4
*Mw*	14,327	11,481	12,223	9,807
*Mn*	9,372	7,934	8,515	6,651
*Mw*/*Mn*	1.5	1.4	1.4	1.5
Interunit linkages^a^				
β−O−4′	44.1	41.5	40.4	42.3
β−5′	7.8	9.3	11.2	7.9
β−β′	4.7	5.1	5.6	5.3
Condensed degree^b^	22.1	25.8	29.4	23.8
Aromatic units^c^				
H	9	8	8	9
G	52	52	49	50
S	39	40	43	42
S/G ratio	0.75	0.77	0.88	0.84

^a^ Molar percentage (H + G + S = 100).

^b^ Condensed degree, % = 100 * (IB_α_+ IC_α_)/(IA + IB_α_+ IC_α_), which is referred to as the integral value of each signal in 2D HSQC NMR.

^c^ Interunit linkages molar contents as percentages of lignin content (H + G + S).

### 3.2 Chemical structure characterizations

**FIGURE 3:**
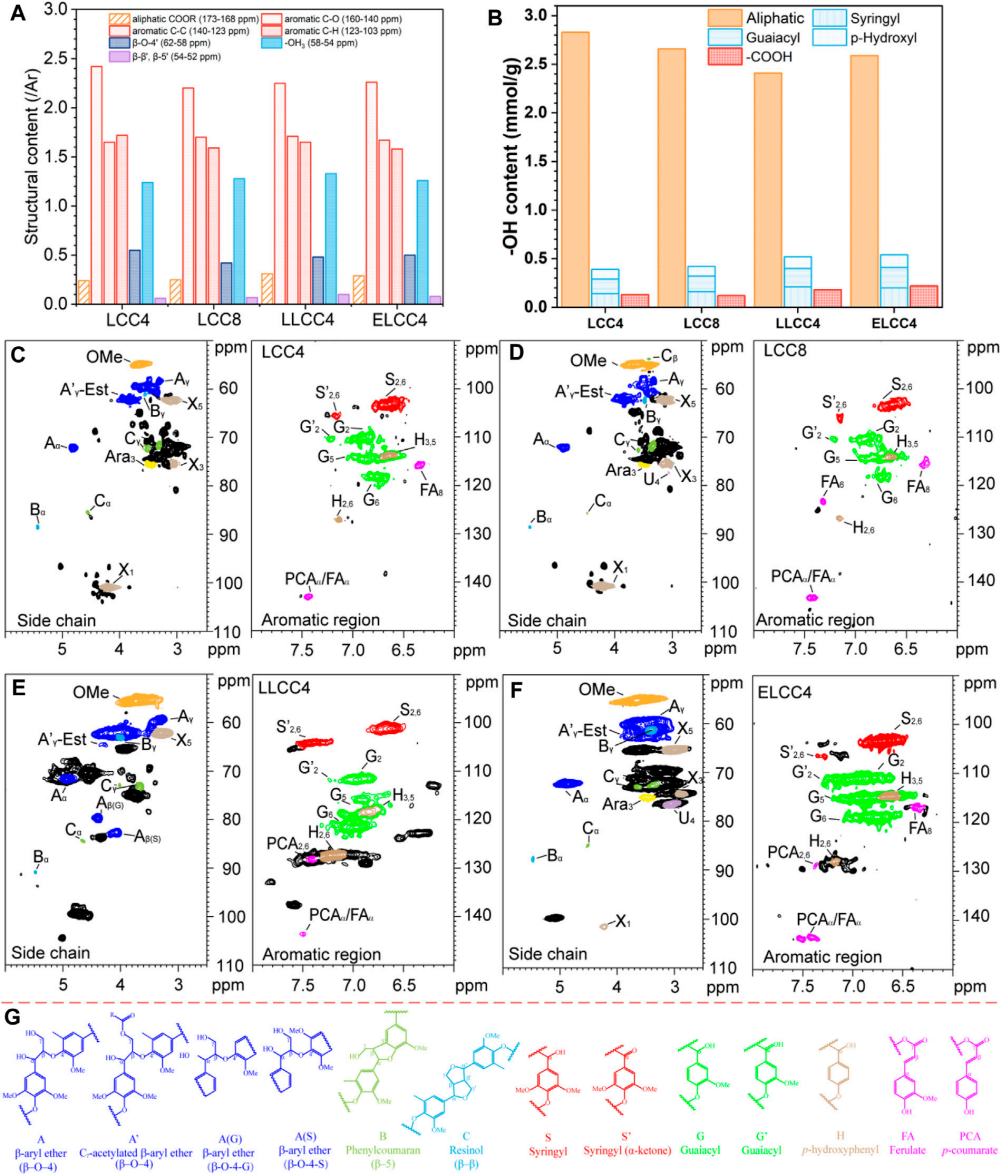
**(A)** Quantitative ^13^C NMR (/Ar), **(B)** quantitative ^31^P NMR for functional groups (mmol/g), 2D HSQC NMR of **(C)** LCC4, **(D)** LCC8, **(E)** LLCC4, and **(F)** ELCC4. **(G)** the internal structures of LCC preparations.

The ^13^C MMR, ^31^P NMR, and 2D NMR were performed to reveal the structure variation of all preparations (Figure 3; Supplementary Figure S1), and the assignment was listed in Supplementary Table S1. All preparations were similar in ^13^C NMR, especially for LCC4 and LCC8. Compared to the nonenzymatic LCCs, the weaker signals of aliphatic -COOR (173–168 ppm) in ELCC4 indicated that an amount of Est bonds was cracked (Figure 3A; Supplementary Figure S1A). The G_6_ chemical shift in LLCC4 was due to the condensation of conjugated structures (C=O) or C_5_ in aromatic rings (Wen et al., 2013), resulting in the oxidized G-unit lignin (C_α_ = O) on side chains and condensed G-unit lignin at 123.6 and 130.8 ppm (Supplementary Figure S1A), respectively.

The lignin solution with phosphitylating regent was shaken at room temperature for 30 min, and then immediately analyzed. The aliphatic -OH content decreased while phenolic -OH and -COOH content increased in LLCC4 (Figure 3F; Supplementary Figure S3C). The increased content of phenolic -OH was due to the β-O-4′ cleavage during the acid depolymerization of LiCl-DMSO/NaOH-H_2_O separation process, and the increased -COOH content was from the oxidation of aliphatic -OH groups (Mohamad Ibrahim et al., 2011). The content of phenolic -OH and -COOH in ELCC4 was greater than that in LCC4 and LCC8, indicating that the partial LC bonds in ELCC4 were enzymatically hydrolyzed, such as PhGlc or γ-ester, releasing more functional groups. Moreover, hydrogen bonds between -OH groups of lignin and carbohydrates were disrupted during the enzymatic hydrolysis, consequently exposing more -OH.

As shown in Figures 3C∼E, all LCCs showed obvious carbohydrate contour signals, e.g., xylan (X), Araban (A), and glucuronic acid (U), and retained relatively intact lignin structures with the β-O-4′ content over 40% (Table 2). The β-O-4 linkages of LLCC4 were slightly lower than that of other LCCs, while the condensed degree of LLCC4 was just 7% greater than that of LCC4 (Table 2). However, compared with nonenzymatic LCC, the signals of carbohydrates from ELCC4 decreased after enzymatic hydrolysis (Figure 3F). Compared to the LCCs (LCC4 and LCC8) from solid-liquid separation, the signals at δ_C_/δ_H_ 106.4/7.22 and δ_C_/δ_H_ 117.4/7.3 from S′_2_,_6_ and G′_2_ were both observed in LLCC4, and was much stronger than that in LCC4 and LCC8. These assignments belonging to C_α_ = O were due to the acidic dehydration of the lignin side chain during dissolution-regeneration (Zhu et al., 2021; Su et al., 2024), leading to increased C=O bonds, and endowing the LLCC4 with excellent bioactivities, especially for stabilizing the radicals or reflecting the UV. Noticeably, the S/G ratio of LLCC4 was greater than that of the LCC4. This was due to that the liquid-liquid separation was performed under a weak acidic condition, where the G unit lignin was less likely to dissolve because of its high branching (Cai et al., 2020a; Cai et al., 2020b; Cai et al., 2023). Compared to LCC4 and LCC8, the higher ratio of S unit lignin in ELCC4 and LLCC4 brought better antioxidant abilities, because S unit lignin was more likely to ionize the phenolic -OH, making it easier to trap free radicals or damage the cell membranes of microbe.

### 3.3 Antioxidant and anti-ultraviolet abilities

**FIGURE 4:**
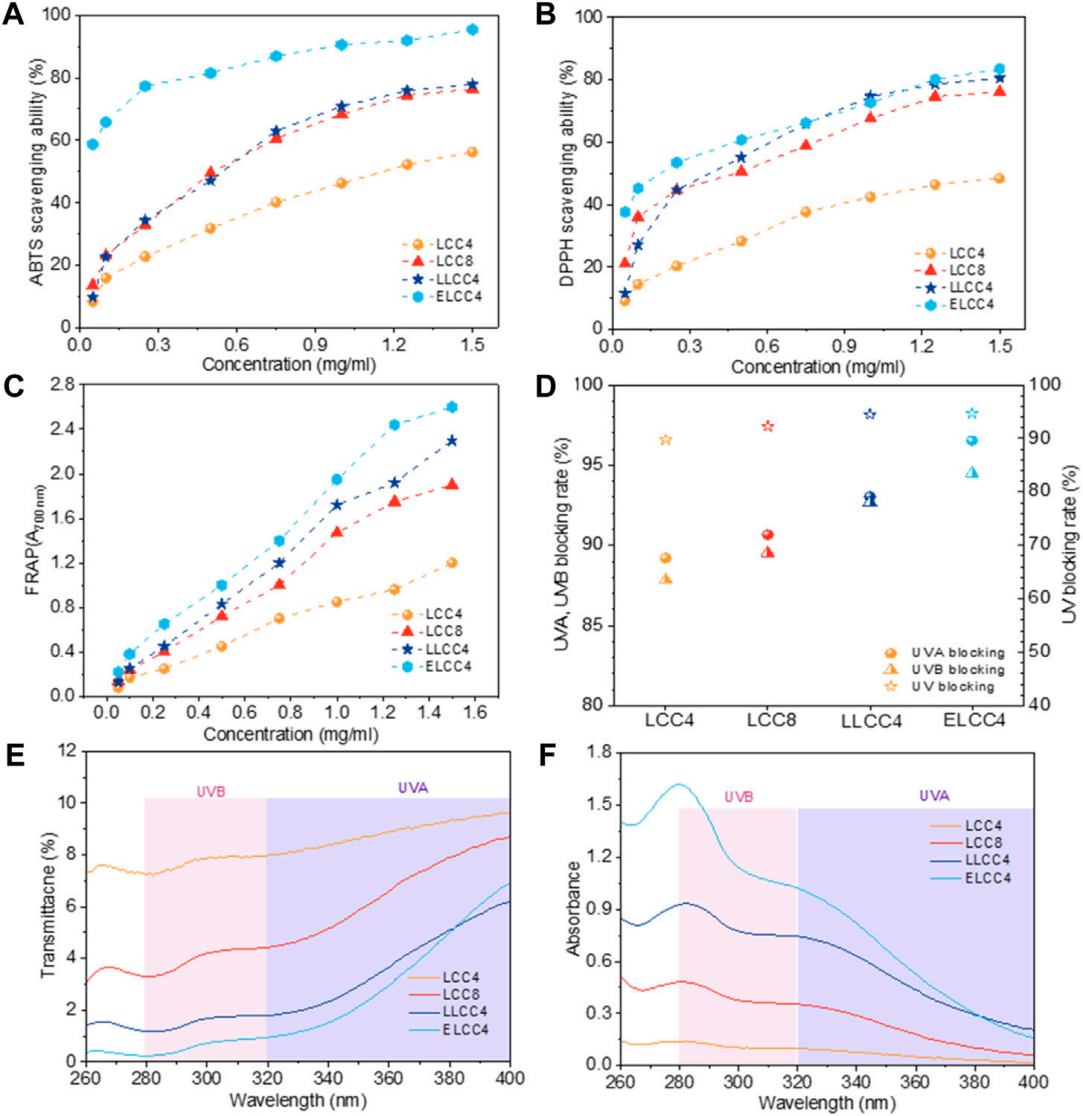
The scavenging ability of LCC preparations toward **(A)** ABTS and **(B)** DPPH radicals, **(C)** Ferric reducing antioxidant power of LCCs (A_700 nm_: the absorbance value at the wavelength of 700 nm), **(D)** the UVA-, UVB-, and UV-blocking rate of LCCs. The UV-vis **(E)** transmission and **(F)** absorbance spectra of LCC samples at a solution concentration of 0.5 mg/mL.

Antioxidant ability is important for LCC as a potential bio-radical scavenger, the DPPH, ABTS radicals, and ferric-reducing antioxidant power (FRAP) were employed for the evaluation. From Figure 4A∼C∼ we knew that all the preparations could eliminate ABTS, and DPPH radicals and reduce the Fe^2+^ with a positive dosage dependence. Notably, the antioxidant ability of nonenzymatic LCCs was lower than that of ELCC4. Moreover, the LLCC4 exhibited better antioxidant activity than that of LCC4 and LCC8, suggesting that the chemical pretreatment had a better influence on enhancing the antioxidant ability of LCC. The highest scavenging rate appeared in ELCC4 was over 90% and 80% toward ABTS and DPPH, respectively, which was increased by 62% and 71% compared with LCC4, indicating that biological pretreatment was the best approach to improve the antioxidant of LCC. This result may be due to that the ELCC4 exhibited distinct structural variations compared to the other LCC samples. Compared to LCC4, ELCC4 significantly removed carbohydrates by enzyme, then exposure of more antioxidant active sites, i.e., phenolic -OH and -COOH, also decreased molecular weight and increased the uniform. More importantly, the bio-functional groups of phenolic -OH and -COOH content were outstandingly improved after biological pretreatment. These factors contribute to the enhanced antioxidant capacity of ELCC4. As reported in previous works (Wang H. et al., 2021; Anushikha, 2023), the phenolic -OH or -COOH could absorb or reflect the UV through phenolic -OH or -COOH conjugated with aromatic structures of lignin. The hydrolysis of polysaccharides in LCC, not only released significant amounts of bioactive groups previously masked and occupied but also resulted in decreased *Mw* and narrower PDI (Saini et al., 2016), both contributing to the enhancement of bioactivities.

The antiultraviolet capacity of LCCs was tested by measuring the UV-vis spectra based on Equations 1–6. In Comparison with LCC4 or LCC8, ELCC4, and LLCC4 had greater abilities in blocking UV-ray at 0.5 mg/mL, which was proved by the low spectral transmittance and the highest UV-blocking rate (94.7%) of ELCC4 in Figures 4D, E. The reason was probably due to the higher phenolic -OH, as well as the C=O (-COOH and carbonyl groups) contents in ELCC4 and LLCC4, which could absorb the photon and convert it to heat simultaneously (Lin et al., 2021). Furthermore, all LCCs exhibited better capacities for blocking the UVA than those for blocking the UVA light. This phenomenon is because the lignin component within LCC is more effective in absorbing UVA, leading to the UVA blocking rate of LCC being higher than the UVB blocking rate. As shown in Figure 4F, the UV-absorption properties of the ELCC4 showed a higher maximum absorption value compared to nonenzymatic LCCs ranging from 280 to 400 nm, especially for the UVB region (280–320 nm), which was high up to 92% for UVB-blocking rate, suggesting that most human cell dangers of UVB ray could be effectively blocked by ELCC4.

## 4 Conclusion

Through the comparison of three pretreatment methods for isolating LCC from wheat straw, we proved that the LCC from chemical or biological pretreatment had superior affection in improving the antioxidant and antiultraviolet capacity than that from physical pretreatment. Particularly, biological pretreatment can maximally remove the carbohydrates from LCC and release the functional groups, while the main structure of LCC could be minimally damaged. Compared to the LCC from physical pretreatment (LCC8), LCCs from chemical and biological pretreatments (LLCC4 and ELCC4) exhibited lower *Mw* and PDI, also better thermal stability. The results demonstrated that the ELCC4 and LLCC4 had more functional groups, i.e., phenolic -OH, -COOH, and C=C bonds, which significantly enhanced the bioactivities of LCC. The highest antioxidant property against ABTS of ELCC4 was increased to 95%, which was 1.7 times of LCC4, and the DPPH elimination rate of ELCC4 was increased by 71% compared to the LCC4. Furthermore, ELCC4 also showed the best UV-blocking property of 96%, which was increased by 6% and 1% compared to LLCC4 and LCC4. The findings from this study have the potential to guide the development of high-value biomass utilization, ultimately contributing to the advancement of green chemistry and bio-economy initiatives.

## Data Availability

The original contributions presented in the study are included in the article/Supplementary Material, further inquiries can be directed to the corresponding authors.

## References

[B1] Anushikha K. K. G. (2023). Lignin as a UV blocking, antioxidant, and antimicrobial agent for food packaging applications. Biomass Convers. Biorefinery 14, 16755–16767. 10.1007/s13399-022-03707-3

[B2] Bao Y. Du Z. Liu X. Liu H. Tang J. Qin C. (2024). Furfural production from lignocellulosic biomass: one-step and two-step strategies and techno-economic evaluation. Green Chem. 26, 6318–6338. 10.1039/d4gc00883a

[B3] Bian H. Chen L. Dong M. Wang L. Wang R. Zhou X. (2021). Natural lignocellulosic nanofibril film with excellent ultraviolet blocking performance and robust environment resistance. Int. J. Biol. Macromol. 166, 1578–1585. 10.1016/j.ijbiomac.2020.11.037 33181218

[B4] Cai C. Hirth K. Gleisner R. Lou H. Qiu X. Zhu J. Y. (2020b). Maleic acid as a dicarboxylic acid hydrotrope for sustainable fractionation of wood at atmospheric pressure and ≤100 °C: mode and utility of lignin esterification. Green Chem. 22 (5), 1605–1617. 10.1039/c9gc04267a

[B5] Cai C. Li J. Hirth K. Huber G. W. Lou H. Zhu J. Y. (2020a). Comparison of two acid hydrotropes for sustainable fractionation of birch wood. ChemSusChem 13 (17), 4649–4659. 10.1002/cssc.202001120 32463990

[B6] Cai C. Li N. Liu H. Zhang J. Zhu J. Y. Wang F. (2023). Extracting high β-O-4 content lignin and by-producing substrate susceptible to enzymatic hydrolysis by a green flow through process. Chem. Eng. J., 453–139730. 10.1016/j.cej.2022.139730

[B7] Chen L. Dou J. Ma Q. Li N. Wu R. Bian H. (2017). Rapid and near-complete dissolution of wood lignin at ≤80°C by a recyclable acid hydrotrope. Sci. Adv. 3 (9), e1701735. 10.1126/sciadv.1701735 28929139 PMC5600535

[B8] Du X. Gellerstedt G. Li J. (2013a). Universal fractionation of lignin-carbohydrate complexes (LCCs) from lignocellulosic biomass: an example using spruce wood. Plant J. 74 (2), 328–338. 10.1111/tpj.12124 23332001 PMC4091893

[B9] Du X. Li J. Gellerstedt G. Rencoret J. Del Rio J. C. Martinez A. T. (2013b). Understanding pulp delignification by laccase-mediator systems through isolation and characterization of lignin-carbohydrate complexes. Biomacromolecules 14 (9), 3073–3080. 10.1021/bm4006936 23841747

[B10] Gan T. Zhou Q. Su C. Xia J. Xie D. Liu Z. (2021). Efficient isolation of organosolv lignin-carbohydrate complexes (LCC) with high antioxidative activity via introducing LiCl/DMSO dissolving. Int. J. Biol. Macromol. 181, 752–761. 10.1016/j.ijbiomac.2021.03.167 33798581

[B11] Giummarella N. Pu Y. Ragauskas A. J. Lawoko M. (2019). A critical review on the analysis of lignin carbohydrate bonds. Green Chem. 21 (7), 1573–1595. 10.1039/c8gc03606c

[B12] Gu F. Wu W. Wang Z. Yokoyama T. Jin Y. Matsumoto Y. (2015). Effect of complete dissolution in LiCl/DMSO on the isolation and characteristics of lignin from wheat straw internode. Industrial Crops Prod. 74, 703–711. 10.1016/j.indcrop.2015.06.002

[B13] Huang C. Wang X. Liang C. Jiang X. Yang G. Xu J. (2019). A sustainable process for procuring biologically active fractions of high-purity xylooligosaccharides and water-soluble lignin from Moso bamboo prehydrolyzate. Biotechnol. Biofuels 12, 189. 10.1186/s13068-019-1527-3 31384296 PMC6661736

[B14] Jiang B. Zhang Y. Guo T. Zhao H. Jin Y. (2018). Structural characterization of lignin and lignin-carbohydrate complex (LCC) from ginkgo shells (ginkgo biloba L.) by comprehensive NMR spectroscopy. Polym. (Basel) 10 (7), 736. 10.3390/polym10070736 PMC6404004 30960661

[B15] Liang M. Deng J. Gu J. Yang J. Ge F. Huang C. (2023). TMBPF-induced neurotoxicity and oxidative stress in zebrafish larvae: impacts on central nervous system development and dopamine neurons. Ecotoxicol. Environ. Saf. 268, 115710. 10.1016/j.ecoenv.2023.115710 38000302

[B16] Lin M. Yang L. Zhang H. Xia Y. He Y. Lan W. (2021). Revealing the structure-activity relationship between lignin and anti-UV radiation. Industrial crops Prod. 174, 114212. 10.1016/j.indcrop.2021.114212

[B17] Liu Z. Meng L. Chen J. Cao Y. Wang Z. Ren H. (2016). The utilization of soybean straw III: isolation and characterization of lignin from soybean straw. Biomass Bioenergy 94, 12–20. 10.1016/j.biombioe.2016.07.021

[B18] Mohamad Ibrahim M. N. Zakaria N. Sipaut C. S. Sulaiman O. Hashim R. (2011). Chemical and thermal properties of lignins from oil palm biomass as a substitute for phenol in a phenol formaldehyde resin production. Carbohydr. Polym. 86 (1), 112–119. 10.1016/j.carbpol.2011.04.018

[B19] Narron R. H. Chang H.-m. Jameel H. Park S. (2017). Soluble lignin recovered from biorefinery pretreatment hydrolyzate characterized by lignin–carbohydrate complexes. ACS Sustain. Chem. and Eng. 5 (11), 10763–10771. 10.1021/acssuschemeng.7b02716

[B20] Niu H. Song D. Mu H. Zhang W. Sun F. Duan J. (2016). Investigation of three lignin complexes with antioxidant and immunological capacities from Inonotus obliquus. Int. J. Biol. Macromol. 86, 587–593. 10.1016/j.ijbiomac.2016.01.111 26845476

[B21] Saini J. K. Patel A. K. Adsul M. Singhania R. R. (2016). Cellulase adsorption on lignin: a roadblock for economic hydrolysis of biomass. Renew. Energy 98, 29–42. 10.1016/j.renene.2016.03.089

[B22] Sakagami H. Kushida T. Oizumi T. Nakashima H. Makino T. (2010). Distribution of lignin-carbohydrate complex in plant kingdom and its functionality as alternative medicine. Pharmacol. Ther. 128 (1), 91–105. 10.1016/j.pharmthera.2010.05.004 20547183

[B23] Singh R. Singh S. Trimukhe K. D. Pandare K. V. Bastawade K. B. Gokhale D. V. (2005). Lignin–carbohydrate complexes from sugarcane bagasse: preparation, purification, and characterization. Carbohydr. Polym. 62 (1), 57–66. 10.1016/j.carbpol.2005.07.011

[B24] Su C. Gan T. Liu Z. Chen Y. Zhou Q. Xia J. (2021a). Enhancement of the antioxidant abilities of lignin and lignin-carbohydrate complex from wheat straw by moderate depolymerization via LiCl/DMSO solvent catalysis. Int. J. Biol. Macromol. 184, 369–379. 10.1016/j.ijbiomac.2021.06.063 34126153

[B25] Su C. Hirth K. Liu Z. Cao Y. Zhu J. Y. (2021b). Maleic acid hydrotropic fractionation of wheat straw to facilitate value‐added multi‐product biorefinery at atmospheric pressure. GCB Bioenergy 13 (9), 1407–1424. 10.1111/gcbb.12866

[B26] Su C. Hirth K. Liu Z. Cao Y. Zhu J. Y. (2021c). Acid hydrotropic fractionation of switchgrass at atmospheric pressure using maleic acid in comparison with p-TsOH: advantages of lignin esterification. Industrial Crops Prod. 159, 113017. 10.1016/j.indcrop.2020.113017

[B27] Su C. Wang X. Deng Y. Wu T. Jiao J. Huang C. (2024). Revitalizing multiple bioactivities of the lignin-carbohydrate complex through a two-step strategy: unraveling the enhancement origin from a structural variation perspective. ACS Sustain. Chem. and Eng. 12 (28), 10544–10554. 10.1021/acssuschemeng.4c03159

[B28] Tarasov D. Leitch M. Fatehi P. (2018). Lignin-carbohydrate complexes: properties, applications, analyses, and methods of extraction: a review. Biotechnol. Biofuels 11, 269. 10.1186/s13068-018-1262-1 30288174 PMC6162904

[B29] Ullah I. Chen Z. Xie Y. Khan S. S. Singh S. Yu C. (2022). Recent advances in biological activities of lignin and emerging biomedical applications: a short review. Int. J. Biol. Macromol. 208, 819–832. 10.1016/j.ijbiomac.2022.03.182 35364209

[B30] Wang H. Tang X. Arvanitis M. A. Yang V. Stark N. Liu C. (2021b). Colloidal lignin nanoparticles from acid hydrotropic fractionation for producing tough, biodegradable, and UV blocking PVA nanocomposite. Industrial Crops Prod. 168, 113584. 10.1016/j.indcrop.2021.113584

[B31] Wang R. Yan B. Yin Y. Wang X. Wu M. Wen T. (2024a). Polysaccharides extracted from larvae of *Lucilia sericata* ameliorated ulcerative colitis by regulating the intestinal barrier and gut microbiota. Int. J. Biol. Macromol. 270, 132441. 10.1016/j.ijbiomac.2024.132441 38761897

[B32] Wang X. Huang C. Fu X. Jeon Y. J. Mao X. Wang L. (2023). Bioactivities of the popular edible Brown seaweed sargassum fusiforme: a review. J. Agric. Food Chem. 71 (44), 16452–16468. 10.1021/acs.jafc.3c05135 37876153

[B33] Wang X. Huang C. Yang F. Wang K. Cha S. H. Mao X. (2024b). Fucoidan isolated from the edible seaweed Sargassum fusiforme suppresses skin damage stimulated by airborne particulate matter. Algal Res. 77, 103339. 10.1016/j.algal.2023.103339

[B34] Wang X. Qu Y. Jiao L. Bian H. Wang R. Wu W. (2021a). Boosting the thermal conductivity of CNF-based composites by cross-linked lignin nanoparticle and BN-OH: dual construction of 3D thermally conductive pathways. Compos. Sci. Technol. 204, 108641. 10.1016/j.compscitech.2020.108641

[B35] Wang X. Sun M. Wang R. Jiao L. Bian H. Dai H. (2022). Promoting h-BN dispersion in cellulose-based composite by lignosulfonate for regulatable effectual thermal management. Mater. and Des. 214, 110379. 10.1016/j.matdes.2021.110379

[B36] Wen J. L. Sun S. L. Xue B. L. Sun R. C. (2013). Quantitative structures and thermal properties of birch lignins after ionic liquid pretreatment. J. Agric. Food Chem. 61 (3), 635–645. 10.1021/jf3051939 23265413

[B37] Xie D. Gan T. Su C. Han Y. Liu Z. Cao Y. (2020). Structural characterization and antioxidant activity of water-soluble lignin-carbohydrate complexes (LCCs) isolated from wheat straw. Int. J. Biol. Macromol. 161, 315–324. 10.1016/j.ijbiomac.2020.06.049 32531357

[B38] You T. T. Zhang L. M. Zhou S. K. Xu F. (2015). Structural elucidation of lignin–carbohydrate complex (LCC) preparations and lignin from Arundo donax Linn. Industrial Crops Prod. 71, 65–74. 10.1016/j.indcrop.2015.03.070

[B39] Zhang Y. Naebe M. (2021). Lignin: a review on structure, properties, and applications as a light-colored UV absorber. ACS Sustain. Chem. and Eng. 9 (4), 1427–1442. 10.1021/acssuschemeng.0c06998

[B40] Zhou Z. Liu D. Zhao X. (2021). Conversion of lignocellulose to biofuels and chemicals via sugar platform: an updated review on chemistry and mechanisms of acid hydrolysis of lignocellulose. Renew. Sustain. Energy Rev. 146, 111169. 10.1016/j.rser.2021.111169

[B41] Zhu J. Y. Agarwal U. P. Ciesielski P. N. Himmel M. E. Gao R. Deng Y. (2021). Towards sustainable production and utilization of plant-biomass-based nanomaterials: a review and analysis of recent developments. Biotechnol. ofe Biofuels 14 (1), 114. 10.1186/s13068-021-01963-5 PMC8101122 33957955

