# Supplementary material for: Comprehensive insights of pretreatment strategies on the structures and bioactivities variation of lignin-carbohydrate complexes
Source: Front Bioeng Biotechnol. 2024 Aug 20;12:1465328. doi: 10.3389/fbioe.2024.1465328 (PMC11368791; doi:10.3389/fbioe.2024.1465328)
Supplement: Supplementary file 1 [file DataSheet1.docx]

Supporting data for:

**Comprehensive Insights of Pretreatment Strategies on the Structures and Bioactivities Variation of Lignin-Carbohydrate Complexes**

Chen Su^1,2,3^, Xiu Wang^3,4^ , Yongjun Deng^2,3^, Zhongjian Tian^1^, Guigan Fang^1,2,3, *^, Chen Huang^2,3,*^

^1^ State Key Laboratory of Biobased Material and Green Papermaking, Qilu University of Technology, Shandong Academy of Sciences, Jinan 250353, China

^2^ Institute of Chemical Industry of Forest Products, Chinese Academy of Forestry, Nanjing 210042, China

^3^ Co-Innovation Center for Efficient Processing and Utilization of Forest Resources, Nanjing Forestry University, Nanjing 210037, China

^4^ Key Laboratory of Polymer Chemistry and Physics of Ministry of Education, School of Materials Science and Engineering, Peking University, Beijing 100871, China

^*^ Corresponding author.

*E-mail address*: [huangchen3127@njfu.edu.cn](mailto:huangchen3127@njfu.edu.cn) (C. Huang); [ppfangguigan@163.com](mailto:ppfangguigan@163.com) (G. Fang)

**
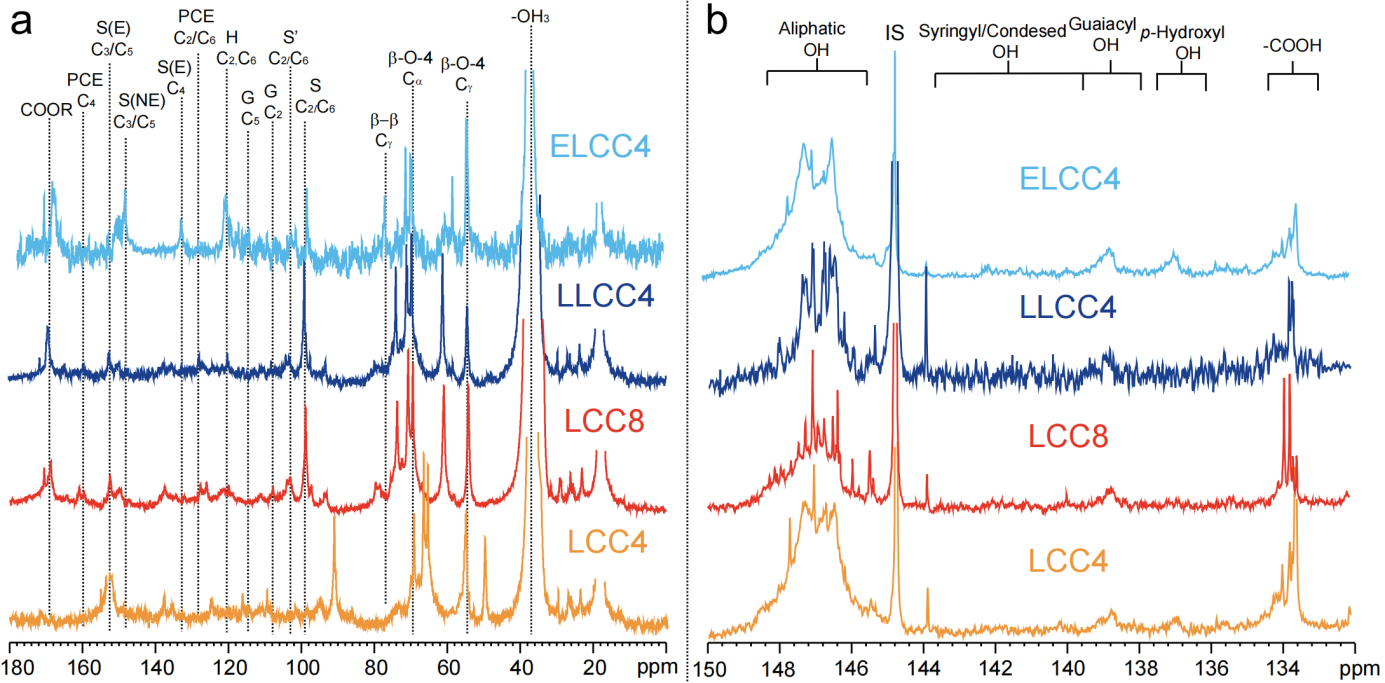
**

**Figure S1.** (a) ^13^C NMR, and (b) ^31^P NMR spectra with quantitative information of different LCCs.

**Table S1.** Assignment of main lignin ^13^C-^1^H cross-signals in the 2D HSQC spectra of different LCCs

| **Labels** | **δ_C_/δ_H_ (ppm)** | **Assignment** |
| --- | --- | --- |
| Lignin structures |  |  |
| C_β_ | 53.7/3.46 | C_β_-H_β_ in phenylcoumarane substructures (C) |
| B_β_ | 54.1/3.06 | C_β_-H_β_ in resinol substructures (B) |
| OMe | 56.2/3.74 | C-H in methoxyls |
| A_γ_ | 60.5/3.61 | C_γ_-H_γ_ in β-O-4' substructures (A) |
| B_γ_ | 63.4/3.70 | C_γ_-H_γ_ in phenylcoumaran substructures (C) |
| A'_γ_-Est | 64.8/4.36 | C_γ_-H_γ_ in γ-acylated β-O-4 substructures (A') |
| C_γ_ | 71.7/4.18 71.7/3.85 | C_γ_-H_γ_ in resinol substructures (B) |
| A_α_ | 72.27/4.82 | C_α_-H_α_in β-O-4' substructures (A) |
| A_β(G/H)_ | 84.1/4.32 | C_β_-H_β_ in β-O-4' substructures linked to G and H units (A) |
| C_α_ | 85.6//4.67 | C_α_-H_α_ in phenylcoumaran substructures (C) |
| A_β(S)_ | 86.7/4.12 87.4/4.02 | C_β_-H_β_ in β-O-4' substructures linked to S units (A) |
| B_α_ | 87.6/5.46 | C_α_-H_α_ in resinol substructures (B) |
| S_2,6_ | 105.5/6.71 | C_2_-H_2_ and C_6_-H_6_ in etherified syringyl units (S) |
| S'_2,6_ | 107.0/7.34 | C_2_-H_2_ and C_6_-H_6_ in syringyl units with C*a*=O groups (S') |
| G_2_ | 111.6/7.01 | C_2_-H_2_ in guaiacyl units (G) |
| G'_2_ | 111.7/7.32 | C_2_-H_2_ in guaiacyl units with C*a*=O groups (G') |
| FA_2_ | 113.1/7.33 | C_2_-H_2_ in ferulate (FA) |
| PCA_β_ | 114.3/6.31 | C_8_-H_8_ in *p*-coumarate (PCA) |
| H_3,5_ | 115.2/6.70 | C_3,5_-H_3,5_ in *p*-hydroxyphenyl units (H) |
| G_5_ | 116.2/6.79 | C_5_-H_5_ in guaiacyl units (G) |
| G_6_ | 119.6/6.81 | C_6_-H_6_ in guaiacyl units (G) |
| FA_6_ | 123.1/7.18 | C_6_-H_6_ in ferulate (FA) |
| H_2,6_ | 128.5/7.23 | C_2,6_-H_2,6_ in *p*-hydroxyphenyl units (H) |
| PCA_2,6_ | 130.7/7.48 | C_2_-H_2_ and C_6_-H_6_ in *p*-coumarate (PCA) |
| FA_α_/PCA_α_ | 145.1/7.49 | C_7_-H_7_ in ferulate (FA) and *p*-coumarate (PCA) |
| Carbohydrate |  |  |
| X_5_ | 63.1/3.22 | C_5_-H_5_ in β-D-xylopyranoside (X) |
| X_2_ | 73.1/3.06 | C_2_-H_2_ in β-D-xylopyranoside (X) |
| X_3_ | 74.6/3.28 | C_3_-H_3_ in β-D-xylopyranoside (X) |
| X_4_ | 75.9/3.52 | C_4_-H_4_ in β-D-xylopyranoside (X) |
| U_4_ | 76.9/3.13 | C_4_-H_4_ in 4-O-methyl-α-D-glucuronic acid (U) |
| Ara_3_ | 77.6/3.69 | C_3_-H_3_ in arabinofuranoside (Ara) |
| X_1_ | 102.3/4.27 | C_1_-H_1_ in β-D-xylopyranoside (X) |
